# Relation between Carious Teeth and Malnutrition

**Published:** 1913-10-15

**Authors:** Charles D. Carter

**Affiliations:** Kingston, N. Y.


### RELATION BETWEEN CARIOUS TEETH AND MALNUTRITION.

BY CHARLES D. CARTER, D.M.D., KINGSTON, N. Y.

Mr. President and members of the Ulster County Medical Association:—I am honored and grateful for this opportunity of addressing you and sincerely hope you will not be disappointed in my few remarks. My subject this evening is: "Relation Between Carious Teeth and Malnutrition." The organs disturbed by disease of the teeth and the oral cavity will be those to which they bear the closest relation. It is well known that the teeth sympathize with each other to such an extent that it is sometimes difficult to determine which one and sometimes which jaw is affected. Otitis media may exhibit itself as toothache, while on the other hand pains in the middle ear are very often mere reflexes of odontalgia. The eye sympathizes with the teeth to such an extent as sometimes to exhibit a profuse lachrymal discharge as the accompaniment of toothache, and alveolar abscess has been diagnosed by the condition of the pulse. The otologist and the dentist should be on intimate terms, as mutual consultation is frequently desirable, owing to the intimate relations of organs concerned.

A perfect masticating appartus uncared for, left to the corroding action of germs and fermenting carbohydrate foods, sugars and starches will in time break down and decay. The process of mastication is the beginning of that series of chemical reaction which change raw material into blood and cells. The use of the teeth is to crush and cut food, converting it into pulp. If the teeth are kept clean and in good repair mastication will be more thorough, and a good start made toward proper digestion. Diseased gums and decayed teeth are in many cases the indirect cause of malnutrition, and as malnutrition effects the whole body, the mouth receives its share, and its resistance to destructive processes is lowered.

Improperly masticated food passes to the stomach where the gastric juice can act on the smallest particles only, while the remainder which has only the surface digested passes to the small intestine where the process, complete or incomplete, is repeated. Decayed teeth are incubators of germs, places for toxines, ptomaines and poisons to be manufactured, and inefficient tools to perform a very necessary operation. Sound teeth, properly placed but unclean, harbor and propagate multitudes of micro-organisms. Between twenty and thirty distinct varieties of bacteria have been found in such mouths. The daily secretion of salvia is about forty-eight ounces, but it has no antiseptic properties as jet discovered. The gastric juice through the agency of the hydrochloric acid is capable of destroying most of these micro-organisms taken in the food. During the resting period, between meals, hydrochloric acid is not secreted, but the constant secretion of impregnated saliva is being constantly swallowed so that a continuous ingestion of germs of even low toxic power in time infects the lining membrane of the stomach, and impairs the glands that secrete the hydrochloric acid and pepsin. Other germs may pass into the small intestine where they can be absorbed into the blood stream, infect the kidneys, liver and pancreas or produce toxines of all degrees of intensity. Infection of the kidneys, liver, pancreas or any of the digestive organs interferes with their functions, so that perfectly proper digestible food cannot be completely assimulated, and cannot be in a condition to be built up into cell substance. This results in malnutrition. Food not properly masticated reaches the stomach in a state not sufficiently chewed to be acted on thoroughly by the gastric juice, this may incite gastritis, or passing into the small intestine may cause putrefaction, and constipation, interfering with the proper nutrition of the body cells.

Fermented starches and sugars are a congenial media for the propagation of micro-organisms other than those producing fermentation. The combination of abnormal fermentation with the great variety of toxic germs lays the foundation for such pathological conditions as dyspepsia, gastric catarrh or septic gastritis which interferes with proper nourishing of the body cells. These changes or results are not produced in a day, a week or months; it may take years but they are sure to materialize.

Let us consider a child from five to ten years of age and see what we find in probably seventy-five cases out of one hundred. In the first place he hasn't had proper instruction in keeping his mouth clean, because he has not been to the dentist, or because the dentist has been careless and didn't take time to notice the condition of his mouth, and instruct him in oral hygiene. He, in time, has a serious toothache, and his mother usually brings him to the dentist and this is what he finds: Mouth unclean, breath foul, cavities in six molars, alveolar abscesses on temporary teeth, pus about the sockets, and root-of temporary teeth lying in the soft tissues. There has been a steady flow of pus to the stomach and intestines for weeks, making a good foundation for chronic diseases of all kinds. Such a condition affects the child's mental ability, causing him to be dull and stupid. In order to correct this condition permanently, the mouth should first be put in a sanitary condition by a competent dentist, and you will then find your diseased conditions will be controlled much easier.

Right here let me say something about indiscriminate extracting of temporary teeth. You will agree with me, I am sure, when I say we have both in the dental and medical profession men who will extract children's temporary teeth without thought of the future or present need of them for masticating. The premature extraction of temporary teeth not only interferes with the nutrition of the child but will change the entire occlusion of the permanent teeth and shape of the oral cavity. The temporary teeth should be filled and retained so long as the permanent teeth show no inclination to erupt.

I had a patient last month, a man about forty-five years of age, who had two temporary molars still in place, and in good healthy condition. This is not an unusual case by any means.

The most distressing and destructive lesion of the oral cavity, and one which receives the least attention from the average practitioner of to-day is pyorrhea alveolaris. This disease, which as is well known destroys the sockets of the teeth and causes their loss if not given proper treatment, has a still more serious effect upon the general constitution of the patient affected. A dentist realizes that if the condition is neglected, the patient will sooner or later lose his teeth, but how many practitioners realize the harm that is being done to the system while the pus from the sockets passes down the throat to the stomach, and from there to the intestines to be passed out through the body in the blood stream, causing any number of diseases. Rheumatism and gout may be traced to pyorrhea alveolaris. In an article published in the *Journal of the American Medical Association*, Dr. J. B. Murphy states that every type of non-traumatic joint inflammation is a manifestation of a primary infection in some other portion of the body. He cites a typical case of metastatic arthritis due to alveolar suppuration or pyorrhea alveolaris. Drs. Wirgman and Turner report in London *Lancet* of recent date forty-two cases of rheumatism and gout, in the majority of which they believe pyorrhea alveolaris to have been the direct cause, because a cure of the local conditions was promptly followed by a subsidence of the constitutional symptoms. I could cite more authority similar to the above, but I believe the foregoing cases will suffice to prove the importance of the oral cavity in relation to general health.

A considerable proportion of pharyngeal and tonsilar affections are the direct result of lesions within the oral cavity, brought about by continuity of tissue.

Complications arising from impacted wisdom teeth and their investments are among the most frequent of these. Owing to the lack of development, especially in the length of the body of the lower jaw, frequently there is not sufficient room for the eruption of the tooth, and it becomes imbedded in the tissues, a constant source of irritation. Sometimes the inflammation about it is so intense as to prevent the opening and closing of the mouth; at times there is a breaking down of tissues and suppuration follows. From the initial point of the lesion dark red lines extending down into the pharynx may be observed, and there is a distinct and acute inflammation of the pillar of the fauces with great discomfort and acute pain. Nine out of ten cases of facial neuralgia are caused by diseased teeth. The irritation from caries may be so severe or so long continued that the trunk of the nerve is affected, and its functions are so modified that it remains in a permanent irritable condition. In diagnosing a case of neuralgia, the mouth and teeth should be the first place to look for the touble, and in most cases an exposed pulp, irritation from tartar or rough edges of fillings will be found to be the cause which places the case entirely in the hands of the dentist.

Roots of decayed and devitalized teeth may sometimes penetrate the floor of the antrum of Highmore and if abscessed will discharge into the cavity, becoming a constant source of irritation to the lining membrane, and in time cause a breaking down of the structure of the mucous follicles. In this manner the roots of teeth may undoubtedly be the cause of actual empyema.

I could spend a longer time in showing you the importance of oral hygiene, but I think you will agree with me when I say that a clean mouth and a well-kept set of teeth is very necessary to general health.—*N. Y. State Journal of Medicine.*

